# Supplementary material for: The genomic landscape of relapsed infant and childhood KMT2A-rearranged acute leukemia
Source: Nat Commun. 2025 Oct 8;16:8964. doi: 10.1038/s41467-025-64190-8 (PMC12508131; doi:10.1038/s41467-025-64190-8)
Supplement: Supplementary file 1 — Supplementary Information [file 41467_2025_64190_MOESM1_ESM.pdf]

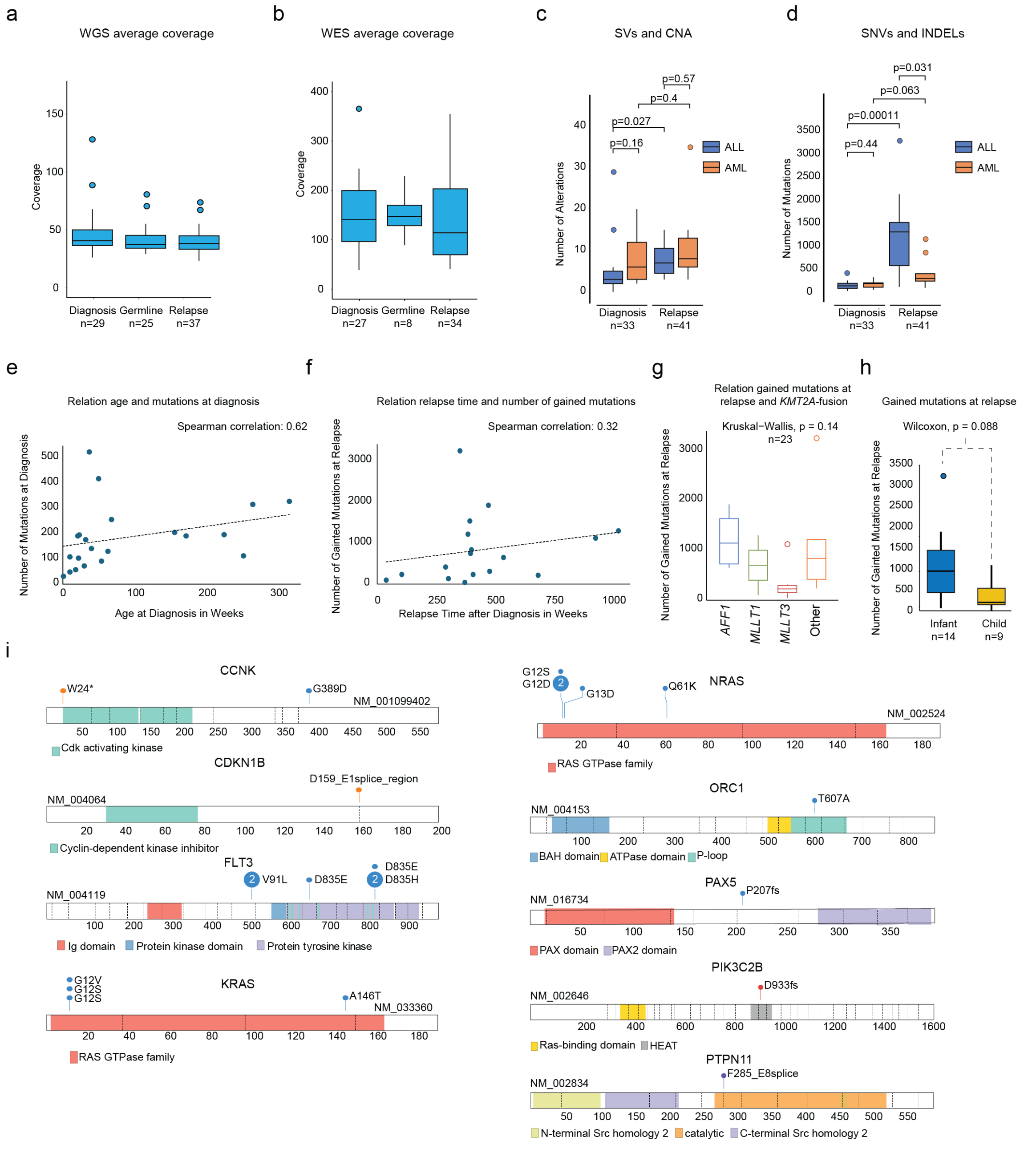

Supplementary Figure 1. Relapse time, sequencing data and mutations in relapse ALL. a, WGS coverage for the diagnostic, germline, and relapse samples. The average is shown by a line. **b,** WES coverage for the diagnostic, germline, and relapse samples. The average is shown by a line and the median by a cross. **c,** Box plots showing the number of unique copy number alterations (CNAs) and structural variations (SVs) in ALL and AML trios at diagnosis and relapse. **d,** Box plots showing the number of mutations at diagnosis and relapse for ALL and AML trios. **e,** Correlation of age at diagnosis (y-axis) and number of mutations at diagnosis (x-axis) for the trios. **f,** Correlation of relapse time (y-axis) and number of mutations at relapse (x-axis) for the trios. **g,** Correlation of gained mutations at relapse and the *KMT2A*-

r for the trios. **h,** Correlation of gained mutations at relapse and the age of the patient for the trios. **i,** Protein paint illustration of mutations identified at relapse, with the x-axis indicating the amino acid position.

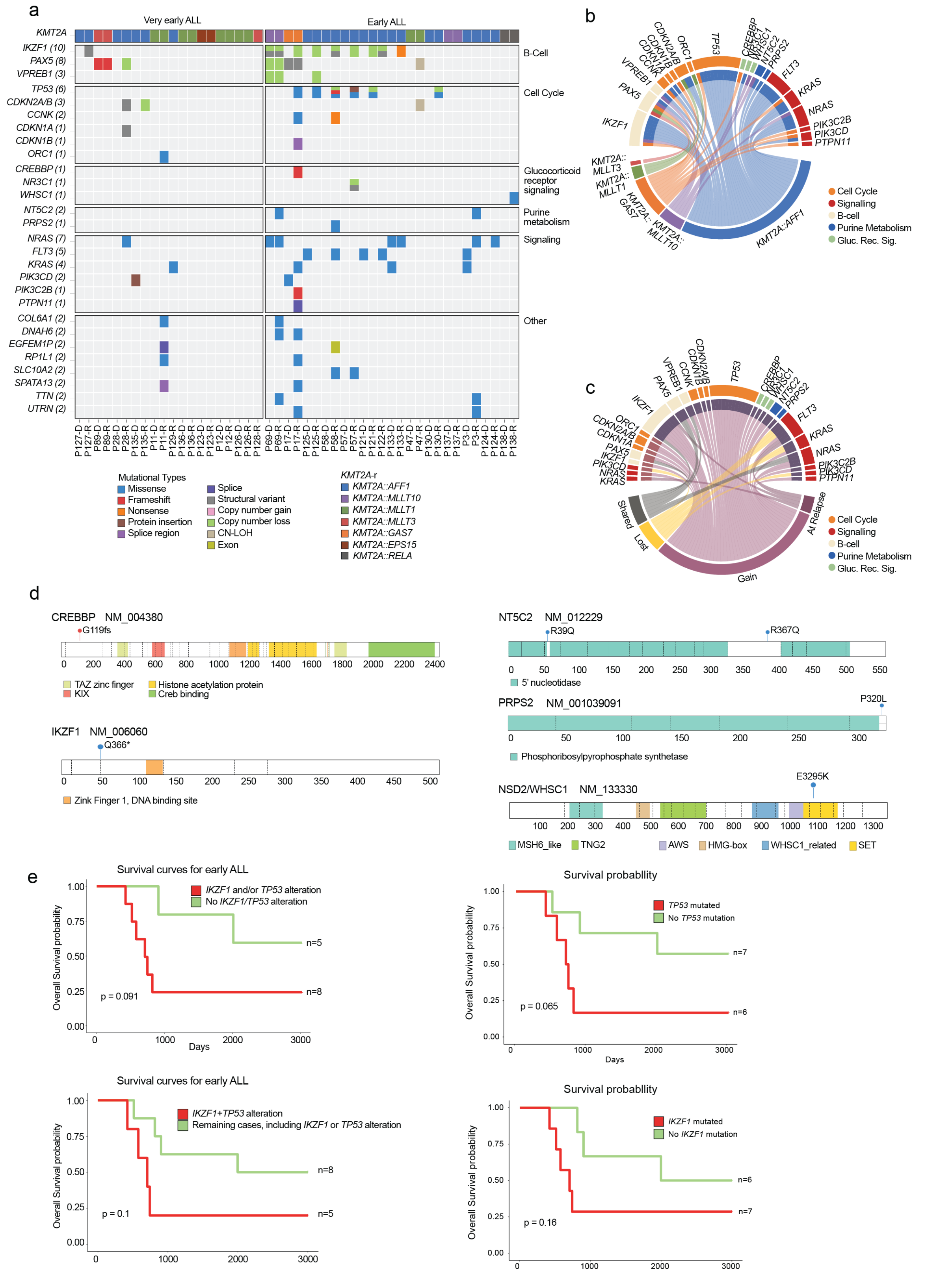

Supplementary Figure 2. Mutations in relapse ALL. a, Heatmap of the relapse ALL cohort showing the specific genetic change that was identified in each gene for the genes in Figure 1d,

as well as other recurrently mutated genes that were not in the recurrent pathways. Diagnostic and relapse samples are next to each other. **b,** Circus plot depicting mutations at relapse for very early and early relapse ALL ordered according to the *KMT2A*-gene rearrangement. **c,** Circus plot depicting mutations at diagnosis (yellow), relapse (purple) and shared (grey) for very early (light purple) and early relapse (dark purple) ALL ordered according to pathway. For patients where only the relapse sample was sequenced, the mutation is shown in dark purple and as "At relapse". **d,** Protein paint illustration of relapse-specific mutations, with the x-axis indicating the amino acid position. **e,** Kaplan Meier curves of the survival probability for early relapse ALL with *IKZF1* and/or *TP53* alterations (n=8) versus those lacking (n=5) at the top left, and below, those with both *IKZF1* and *TP53* (n=5) versus remaining cases (n=8). To the top right, patients with *TP53* versus those lacking and bottom right patients with *IKZF1* versus those lacking. P122 was excluded since survival information was lacking. For figure **e**, source data are provided as a Source Data file.

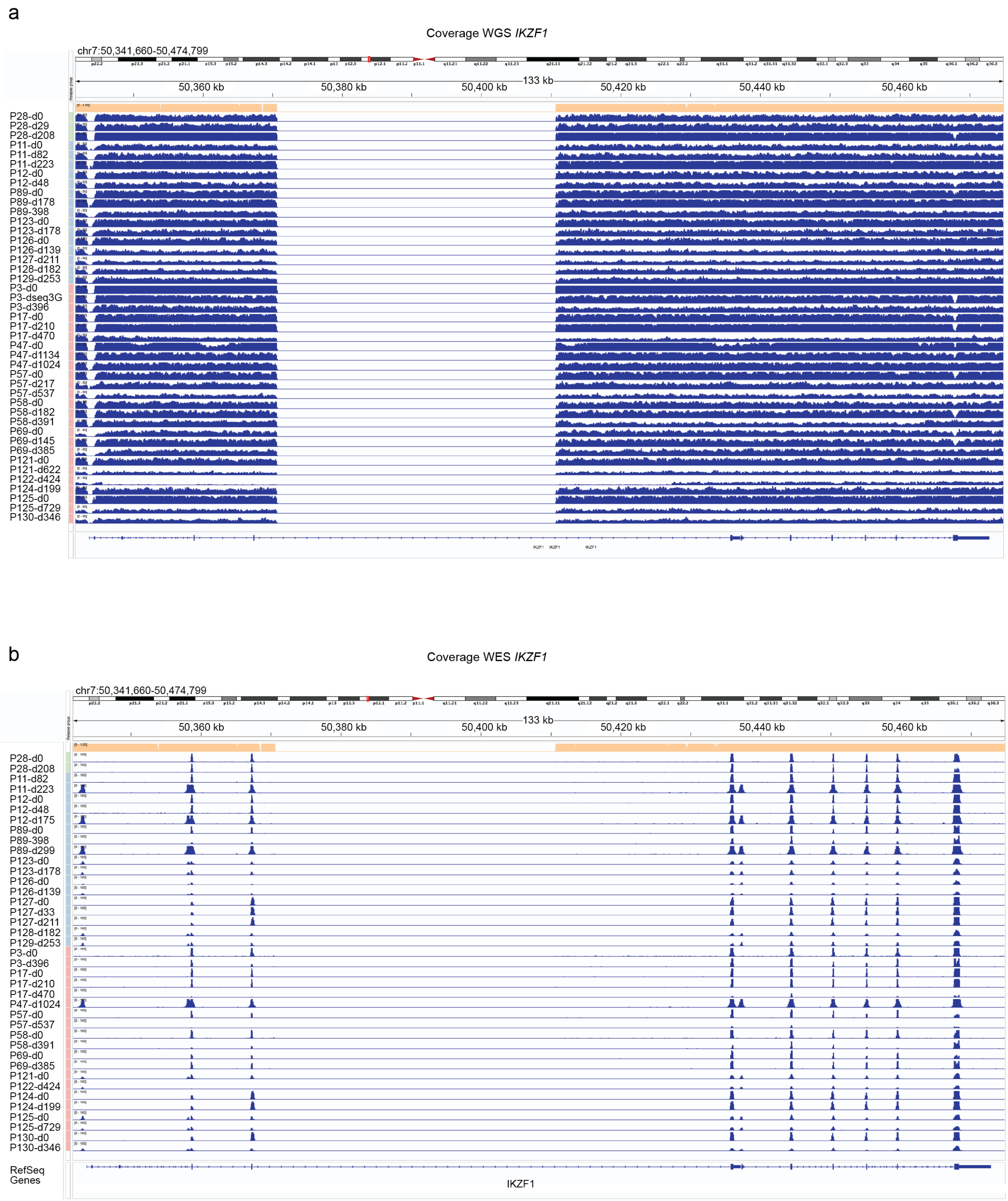

Supplementary Figure 3. WGS and WES coverage for *IKZF1*. a, WGS (whole genome sequencing) coverage for *IKZF1* for the ALL cases (coverage for P135-P138 is available in Supplementary Data 8 only). At the top a schematic illustration of chromosome 7. Each row represents a sample, and the blue bars show the coverage. **b,** As in (**a**) but for WES (whole exome sequencing).

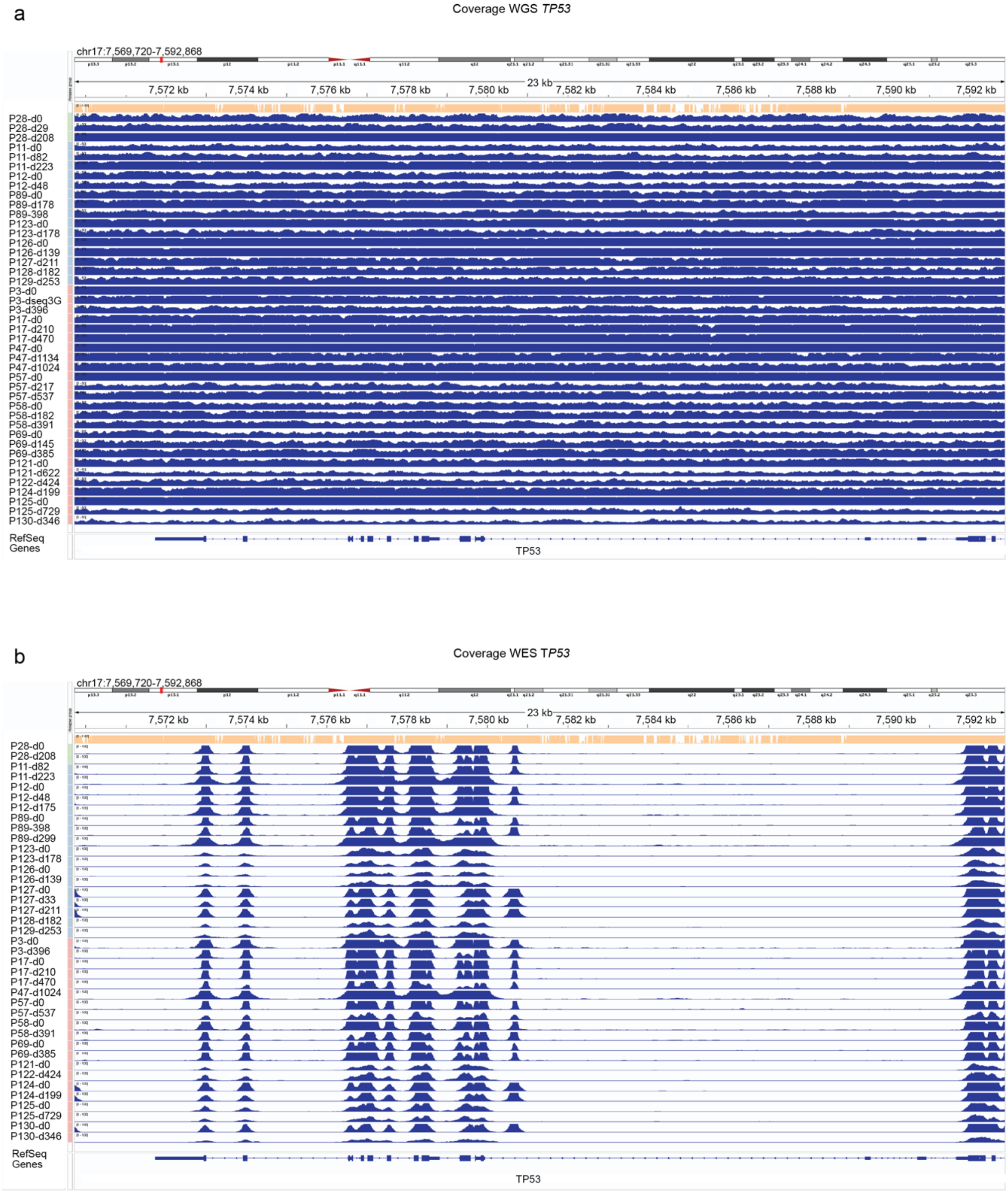

Supplementary Figure 4. WGS and WES coverage for *TP53*. a, WGS coverage for *TP53* for the ALL cases (coverage for P135-P138 is available in Supplementary Data 8 only). At the top a schematic illustration of chromosome 7. Each row represents a sample, and the blue bars show the coverage. **b,** As in (**a**) but for WES.

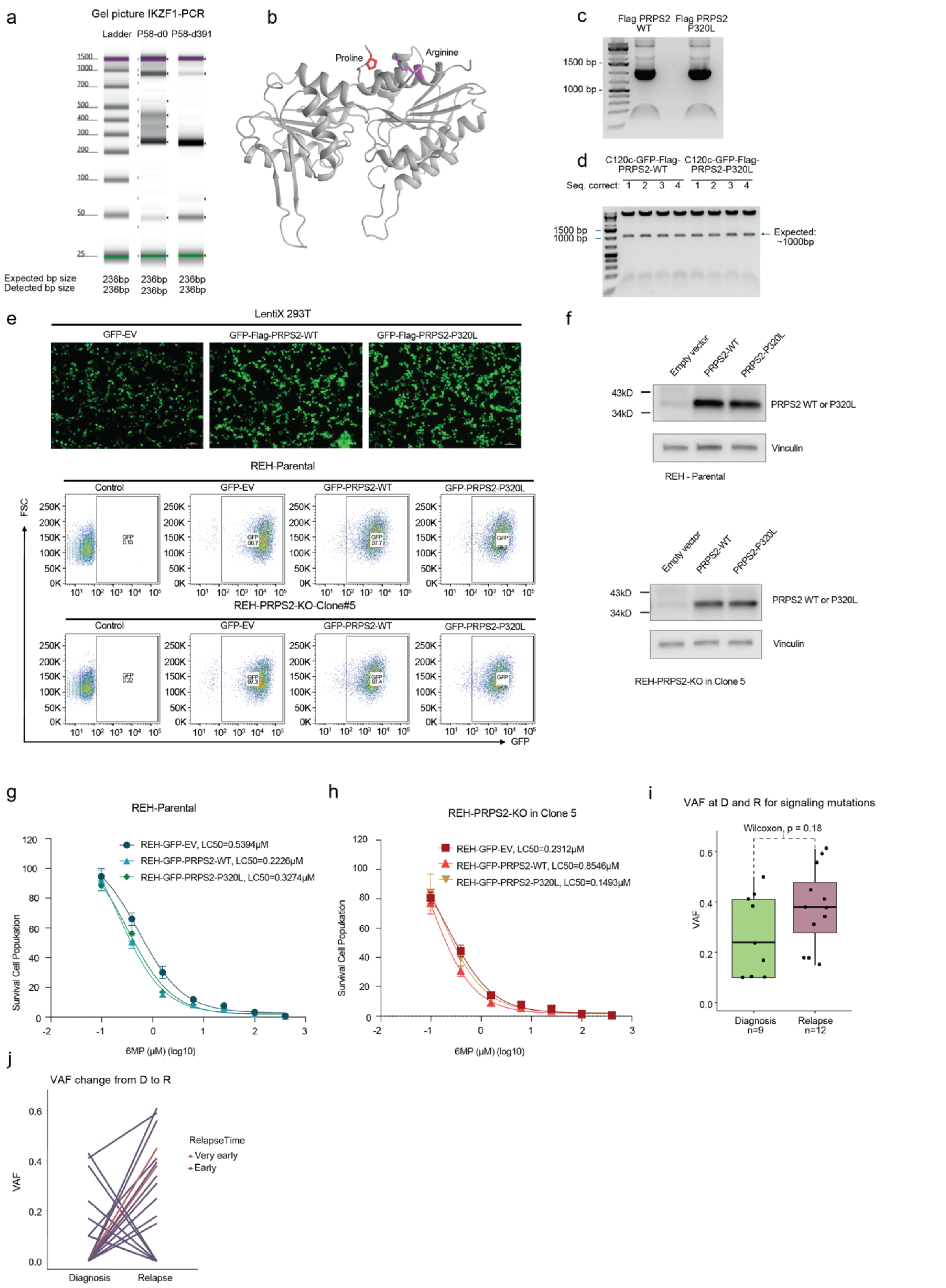

Supplementary Figure 5. ***IKZF1*, assessment of cell viability for PRPS2^{P320L} and VAF of signaling mutations. a,** Tape station data showing the presence of a focal *IKZF1* deletion in

the diagnostic and relapse sample for P58. From left to right, the ladder, the diagnostic sample, and the relapse sample. At the bottom the expected band size and the detected band size are shown. **b,** Alpha Fold model of PRPS2. The proline substitution (P320L) is shown as sticks in red and arginine in lilac (P11908). **c-d**, PCR-amplification of Flag PRPS2 WT and Flag PRPS2^{P320L} following subcloning into cl20c-EF1-GFP. **e**, Lentiviral transduction and infection in the REH cell line, both parental and *PRPS2* KO clone 5. **f**, Western blot of parental REH and in the REH-PRPS2-KO cells. For figure **f**, source data are provided as a Source Data file. **g**, CellTiter-Glo (CTG) assay of the survival in REH parental. **h**, CTG assay of the survival in *PRPS2* KO clone 5. For figures **g** and **h**, source data are provided as a Source Data file. **i**, Boxplot showing the VAF (variant allele frequency) of signaling mutations at diagnosis and relapse in ALL patients, indicating that there is a higher VAF in relapse samples compared to diagnosis. **j,** Graph showing how the signaling mutations' VAFs change from diagnosis to relapse for very early and early relapse patients.

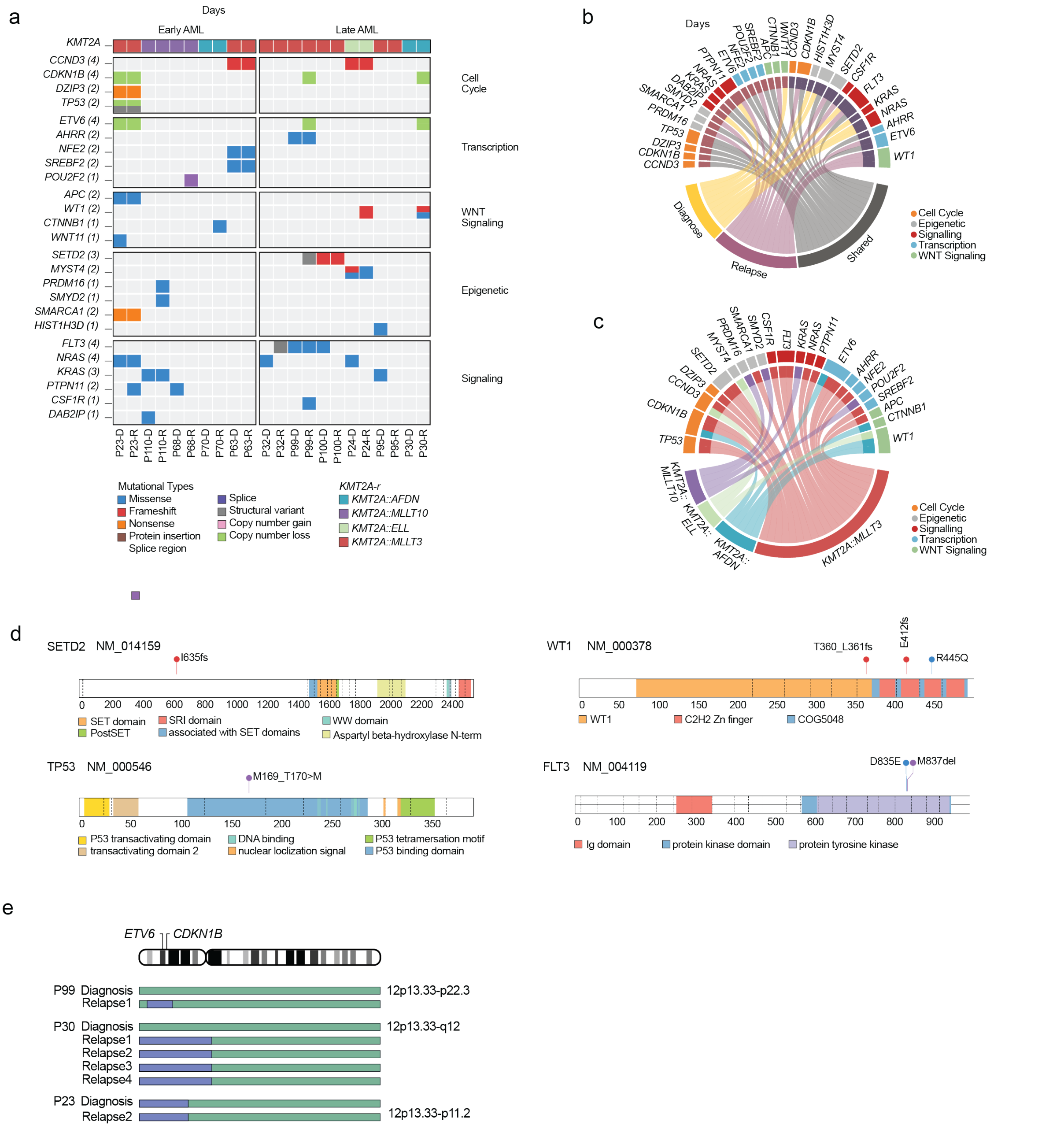

Supplementary Figure 6. Mutational landscape at relapse in AML. a, Heatmap of nonsynonymous mutations in pathways enriched at relapse, with genes in rows and patients in columns. The colors indicate the specific genetic alterations detected at diagnosis and/or relapse in Figure 2b. Diagnostic and relapse samples are next to each other. **b**, Circus plot depicting mutations at diagnosis (yellow), relapse (purple) and shared (grey) for AML patients with early (light purple) and late relapse (dark purple) ordered according to pathway. **c**, Circus plot depicting mutations at relapse for AML patients ordered according to the *KMT2A*-rearrangement. **d**, Protein paint illustration of selected relapse-specific mutations, with the x-

axis indicating the amino acid position. **e,** Illustration of heterozygous 12p deletions at diagnosis and/or relapse.

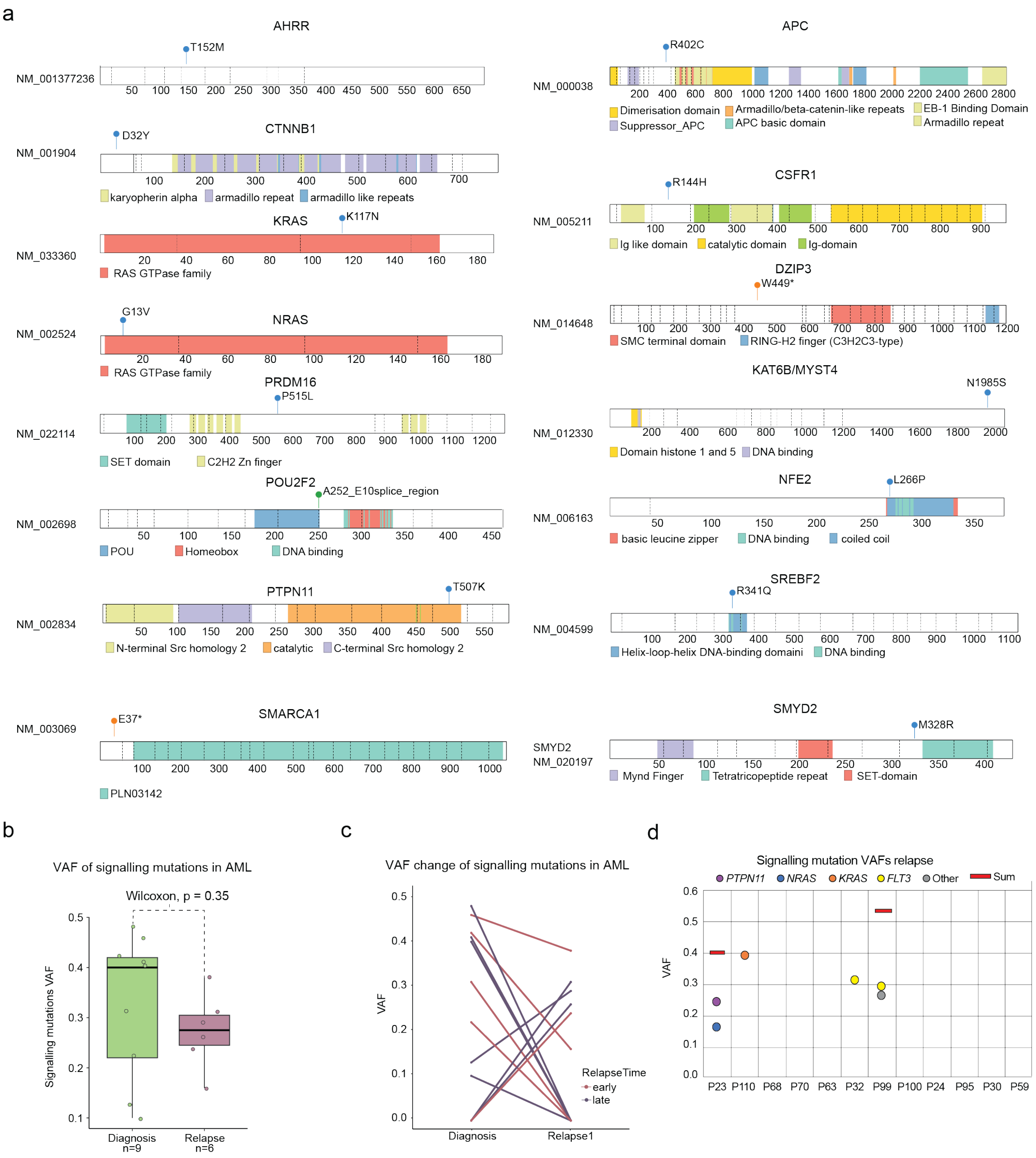

Supplementary Figure 7. Mutational landscape at relapse in AML. a, Protein paint illustration of relapse-specific mutations, with the x-axis indicating the amino acid position. **b,** Boxplot showing the VAF (variant allele frequency) of signaling mutations at diagnosis and relapse in AML. **c,** Graph showing the signaling mutations changes in VAF from diagnosis to relapse for early and late relapse AML. **d**, The VAFs of the signaling mutations at AML relapse.

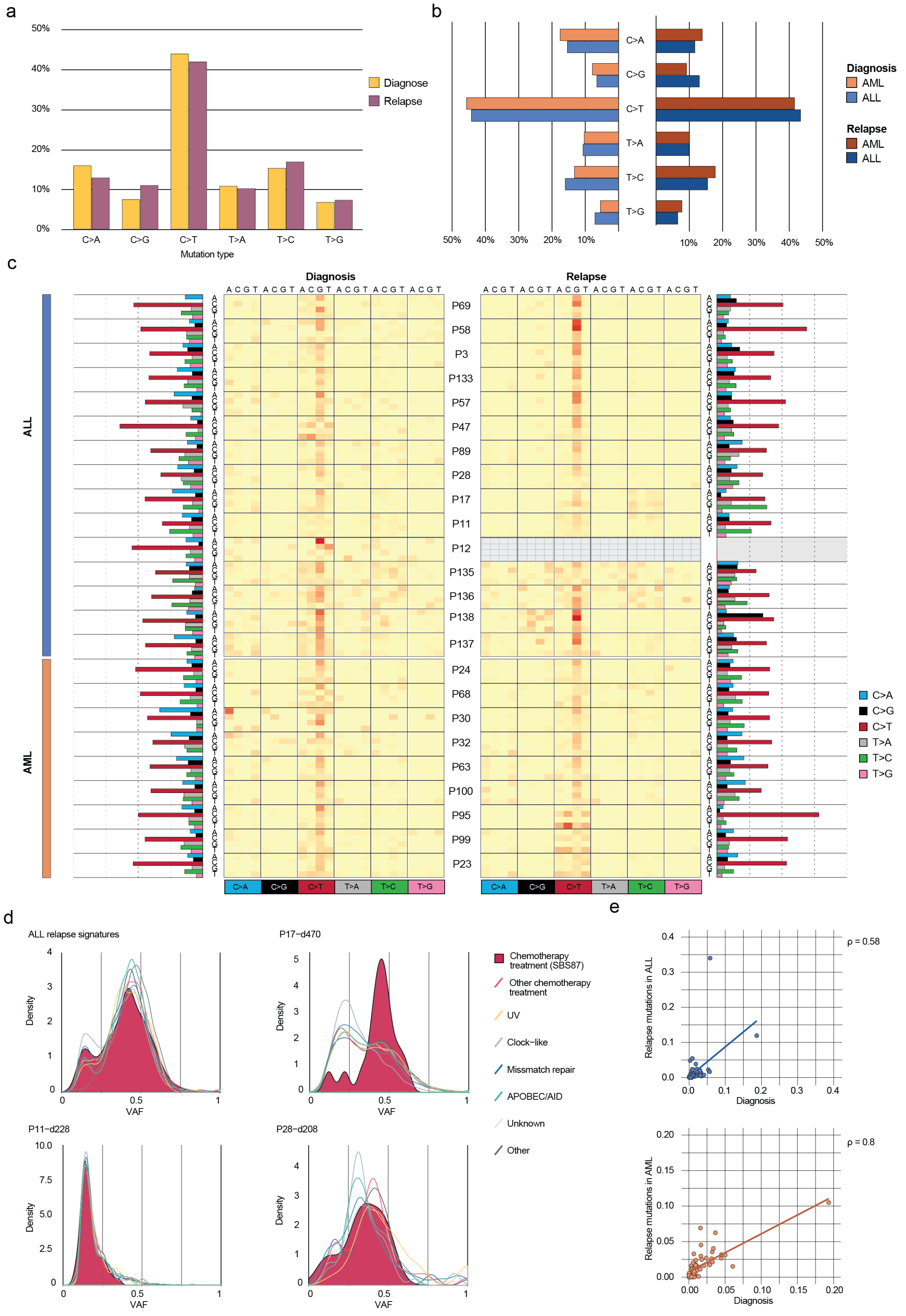

Supplementary Figure 8. Mutational contexts at diagnosis and relapse in ALL and AML.

a, The average distribution of all mutation types per patient from WGS data at diagnosis and

relapse. **b,** The average distribution of mutations per patient from WGS data at diagnosis and relapse in ALL and AML. For figures **a** and **b**, source data are provided as a Source Data file. **c,** The contribution of all possible mutations across the 16 mutational contexts at diagnosis (left) and relapse (right) for the trios that underwent WGS. **d,** Density plot showing the distribution of mutations across the different classes of signatures in all relapse samples (upper left), and P17 (upper right), P11 (lower left), and P28 (lower right), the ALL patients that did not have SBS87 as their primary signature. P17 has an increased VAF in SBS87 signature-related variants, indicative of a clonal expansion of a chemotherapy-affected cell. P11 has mutations from all signatures with low VAFs, and P28 has multiple mutations at high VAFs, i.e., in the dominant clone. The area under the curve is only colored in 'Chemotherapy signature SBS87' for highlighting purposes. **e,** Correlation between the signature contribution of mutations acquired before or after diagnosis in ALL (top) and AML (bottom). In ALL, there is no correlation between diagnosis and relapse ($r^2 = 0.36$). In AML there is a strong Spearman correlation ($r^2 = 0.75$) between diagnosis and relapse. For figure **e**, source data are provided as a Source Data file.

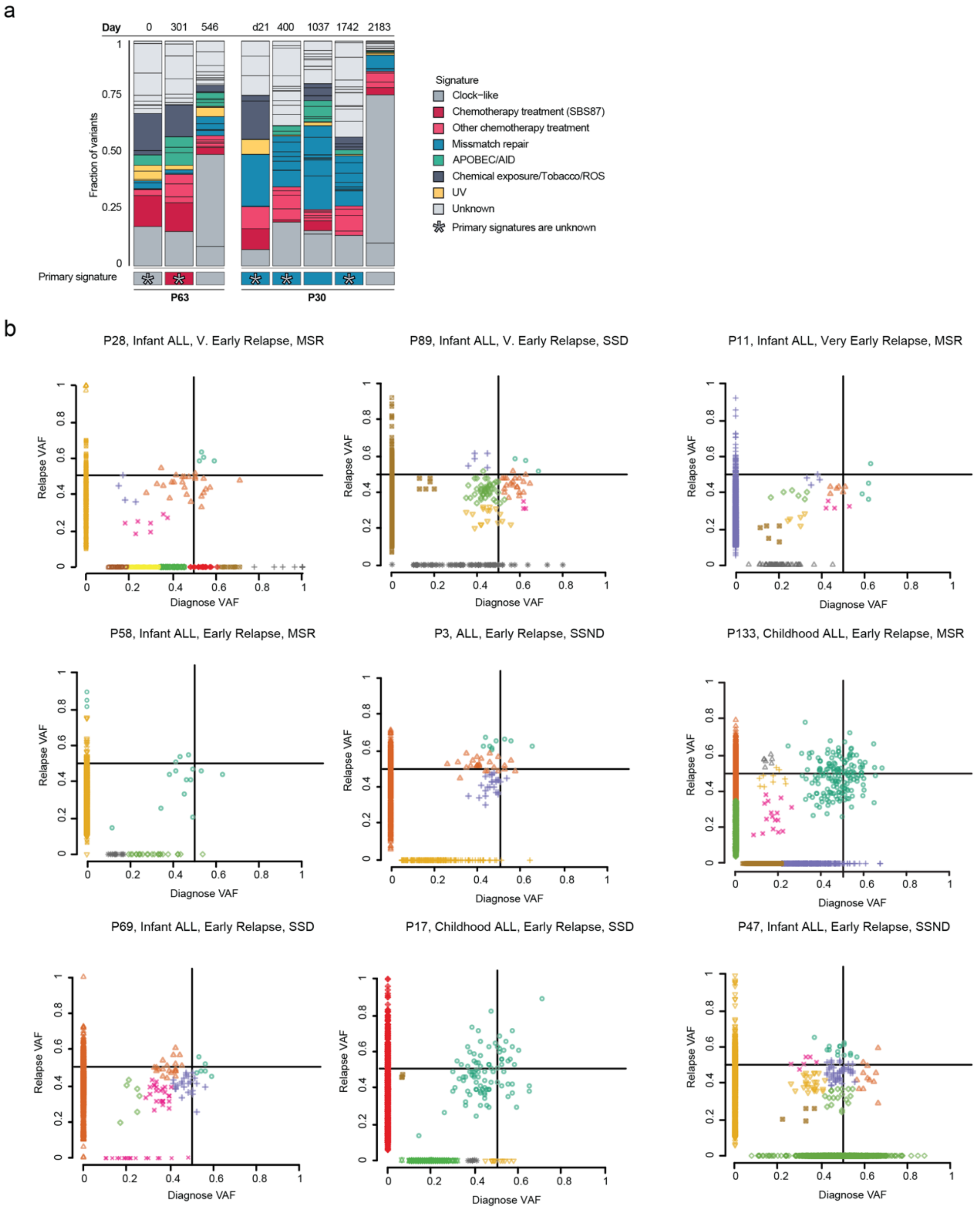

Supplementary Figure 9. Clonal evolution patterns for ALL and AML. a, Mutational signatures in two AML cases with multiple relapses. **b,** 2D-plots with the diagnostic VAF on the x-axis and the relapse VAF on the y-axis. The VAFs are corrected according to the CNAs (copy number alterations). Each dot is represented by a mutation. All relapse samples contained only some of the alterations from the major diagnostic clone, in agreement with relapse arising through branching evolution. Patients were grouped into the following patterns: 1) multiple

clones seed relapse (MSR), or 2) if a sweeping clone was detected at relapse (SSND) and if the sweeping clone was seen already at diagnosis (SSD).

a

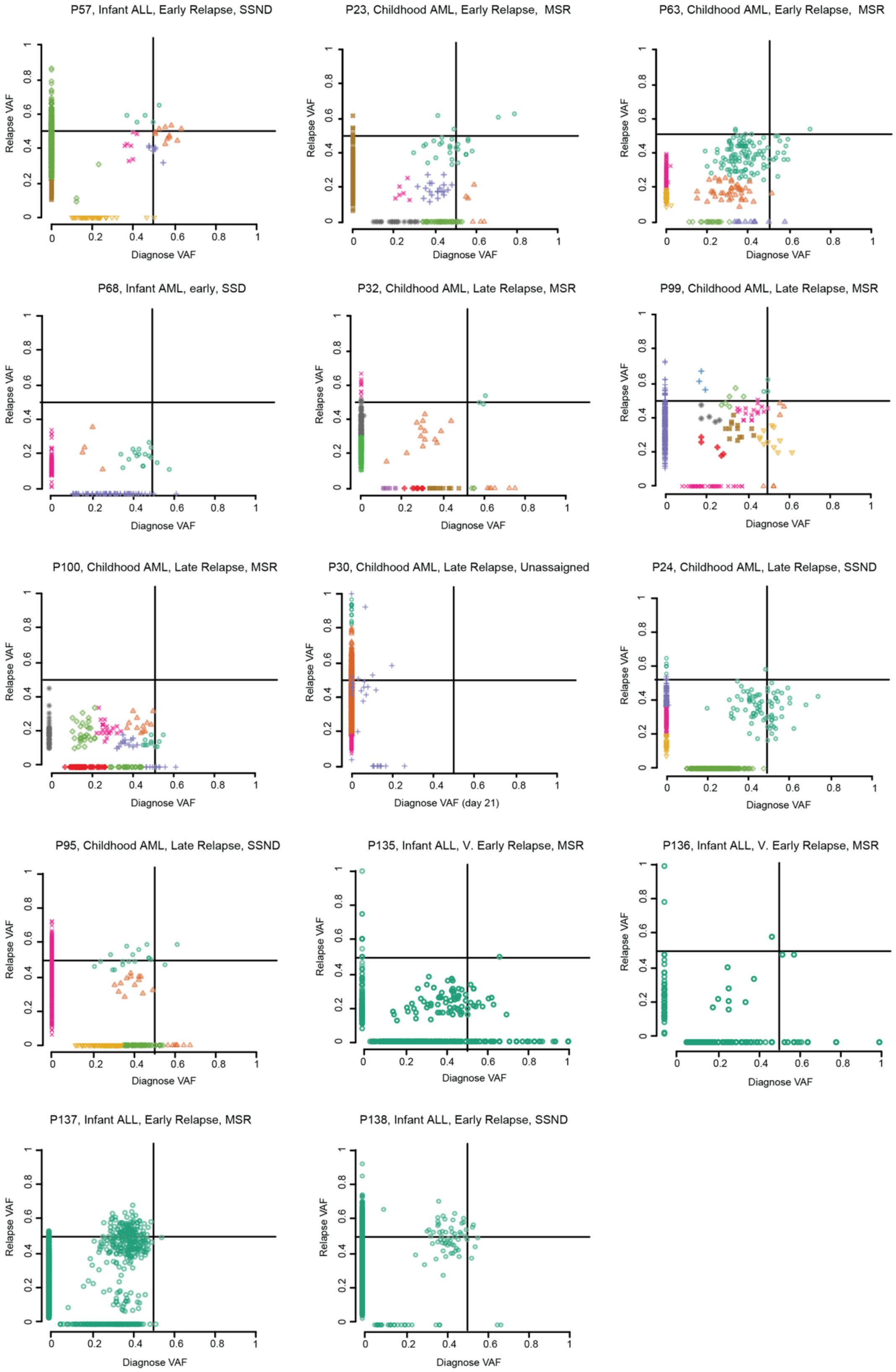

Supplementary Figure 10. Clonal evolution patterns for ALL and AML a. 2D-plots with the diagnostic VAF on the x-axis and the relapse VAF on the y-axis. The VAFs are corrected

according to the CNAs. Each dot is represented by a mutation. All relapse samples contained only some of the alterations from the major diagnostic clone, in agreement with relapse arising through branching evolution. Patients were grouped into the following patterns: 1) multiple clones seed relapse (MSR), or 2) if a sweeping clone was detected at relapse (SSND) and if the sweeping clone was seen already at diagnosis (SSD).

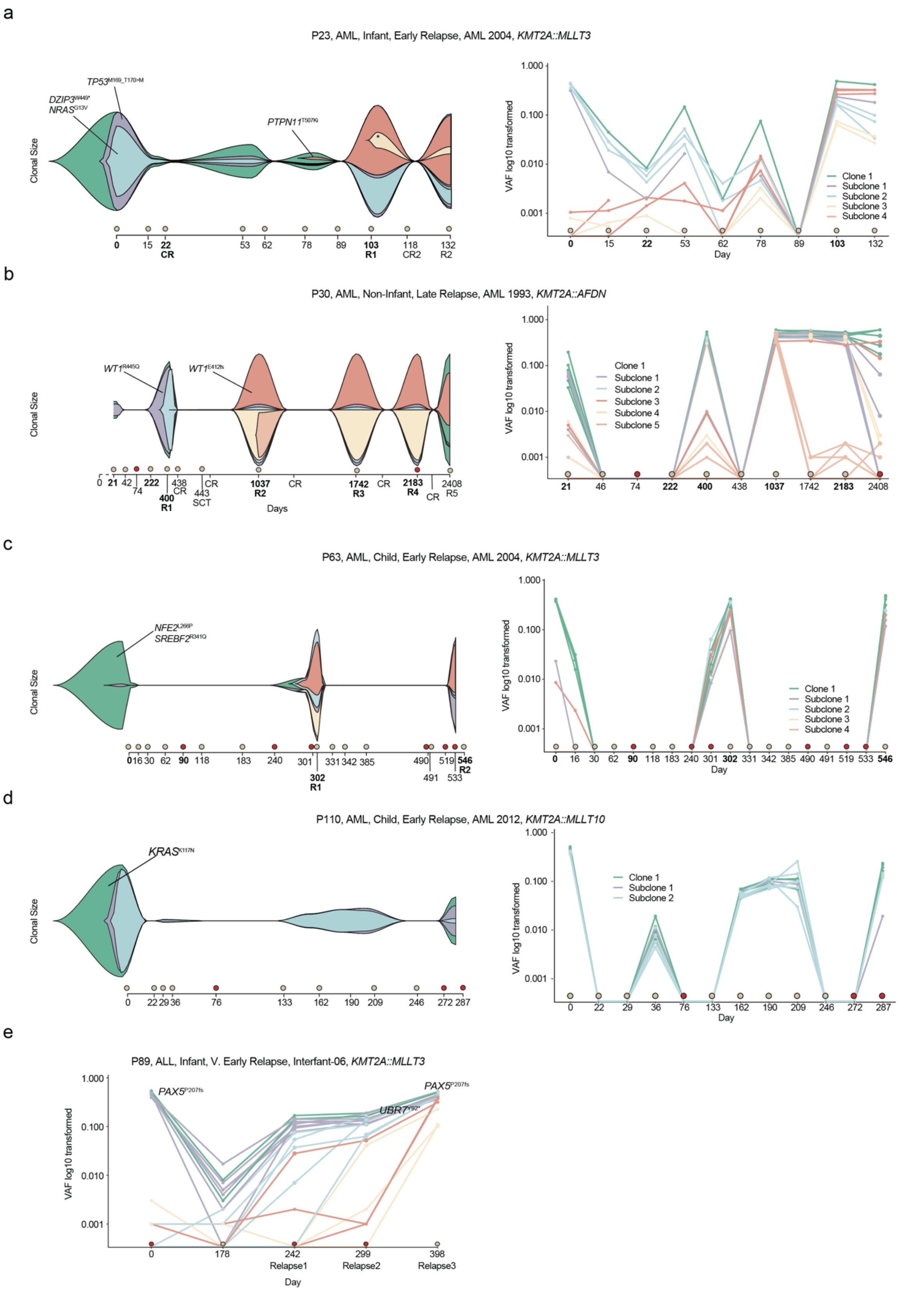

Supplementary Figure 11. The genetic landscape across consecutive relapses. a-d, Clonal evolution by fish plots and linear diagrams for cases with multiple relapses. The VAF (variant

allele frequency) is log10 transformed. Longitudinal BM (beige) and PB (red) samples are on the y-axis, and the clonal size on the x-axis, and clones are in different colors, days in bold indicate WGS. **a,** In this infant AML (P23), the clones expanded after the HA2E1-block, where etoposide was given instead of mitoxantrone, suggesting insensitivity to etoposide and that a new drug-resistant clone (red) containing *PTPN11* expanded, which coexisted with the *NRAS* and *DZIP3*-mutated clone. The patient also had a clonal *APC*$^{\text{R402C}}$ and a subclonal *SMARCA1*$^{\text{E37*}}$ at both diagnosis and relapse and a diagnostic *WNT11*^{R88C} which were not followed longitudinally. **b,** An AML with *KMT2A::MLLT4* (P30), suffered from five relapses over 6 years. Although the patient went into CR by day 21, our data indicated around 15% leukemic cells and molecular remission was not reached until day 42. At the first relapse, a day 21 clone (VAF 0.06) expanded in a sweep (purple) with a *WT1*$^{\text{R554Q}}$, which was maintained until the 5th relapse (VAF 0.06), and a *WT1*$^{\text{V362fs}}$ (not shown) which was lost in subsequent relapses. The patient started on the NOPHO AML-90 relapse protocol with Daunorubicin and FLAG, went into remission and received a BM transplantation. At the second relapse, more than 1000 days after diagnosis, another *WT1*$^{\text{412fs}}$ was detected in 68% of cells and was maintained across the remaining relapses. The patient entered remission and had a third relapse after another two years (day 1742). At the fourth relapse almost 6 years after diagnosis, the relapse was clonally similar to the third relapse with both *WT1*$^{\text{412fs}}$ and *WT1*$^{\text{R554Q}}$ present. At the fifth relapse, only the day 21 clone and the *WT1*$^{\text{412fs}}$-clone (46%) were detected. This highlights how important the *WT1*-mutations were for this leukemia to escape treatment as they survived the therapeutic pressure over several years. **c-d,** Two children with AML and two relapses each, with a clonal change between the first and second relapse. P63 also had a *CCND3*$^{\text{A269fs}}$ at diagnosis and relapse and P110 a *DAB2IP*$^{\text{S58Y}}$-clone lost at relapse and two relapse-specific mutations, a clonal *PRDM16*^{P515L} and a subclonal *SMYD2*^{M328R}, by WGS. **e**, Linear diagram of an infant ALL who had three relapses. The *PAX5* mutation is present in all three relapses. At the second relapse, a *UBR7* mutation is detected, which expands in a sweep at the third relapse.

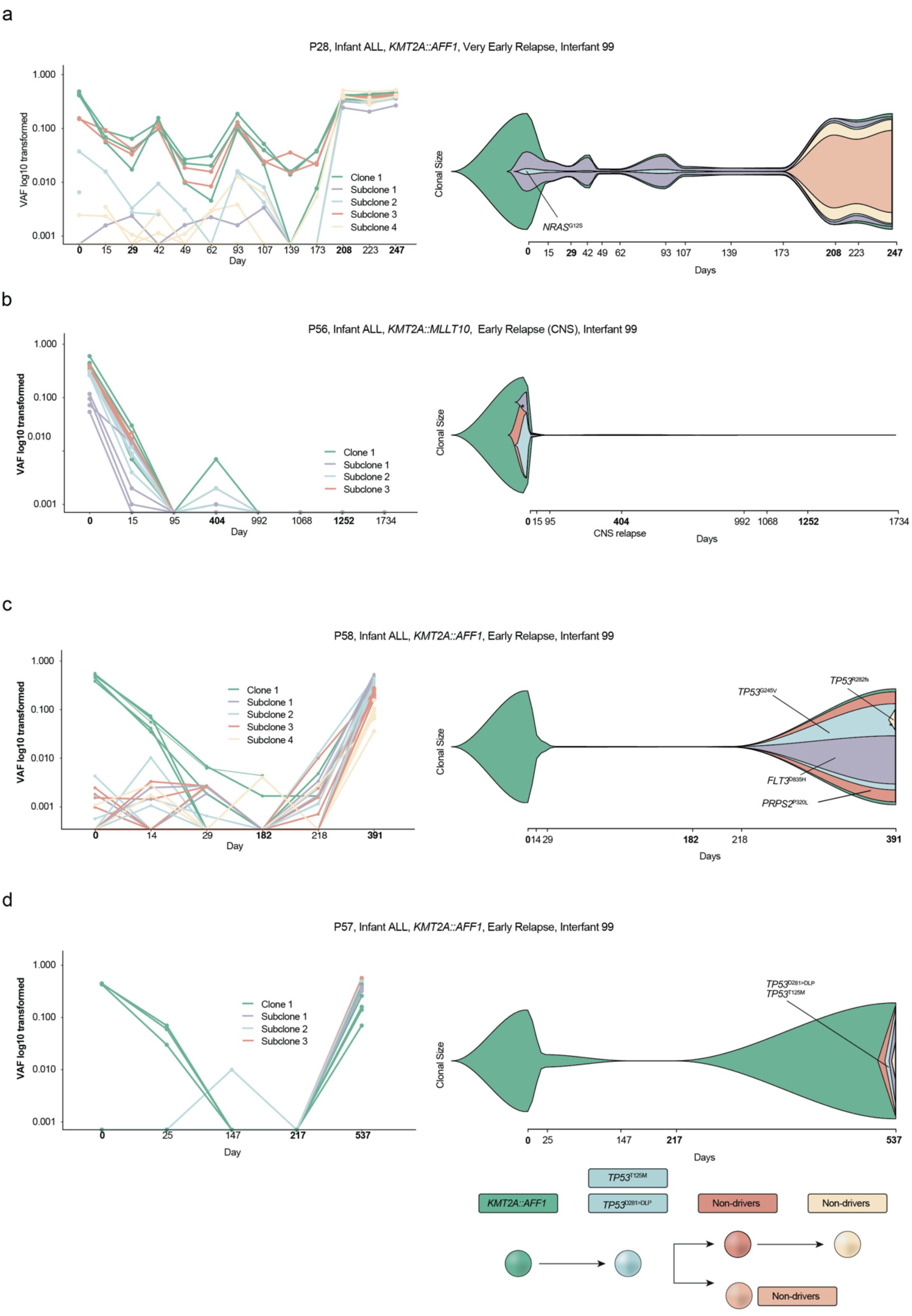

Supplementary Figure 12. Evolution of ALL relapse patients and single-cell DNA sequencing inferring evolution. a-d. Clonal evolution depicted by linear diagrams and fish

plots for relapse patients. Longitudinal samples are on the y-axis and the clonal size on the x-axis. Clones are depicted in different colors. Mutations in the enriched pathways are shown. The VAF is log10 transformed. The * indicates that we cannot determine which clone the subclone belongs to. **a**, Infant with three clones detectable at diagnosis, one of which had an *NRAS*^{G12S} in 2% of the cells. The patient never reached clinical remission and After 208 days, the diagnostic *NRAS*^{G12S}-containing subclone expanded in a selective sweep, and during expansion, it accumulated mutations that were detected at relapse only (yellow and red). Thus, the relapse clone was found in up to 4% of the major clone across treatment and did not expand until the last month of the disease. **b,** Infant with a CNS (central nervous system) relapse at day 404 where we detected 1% of *KMT2A*-r cells in the BM. **c,** An infant with two *TP53*-relapse clones, together with *PRPS2* and *FLT3*-mutations. **d,** A patient that gained two *TP53* mutations at similar VAFs and single-cell DNA-sequencing data inferred the order of acquired mutations and confirmed that the *TP53* mutations were present in the same cell.

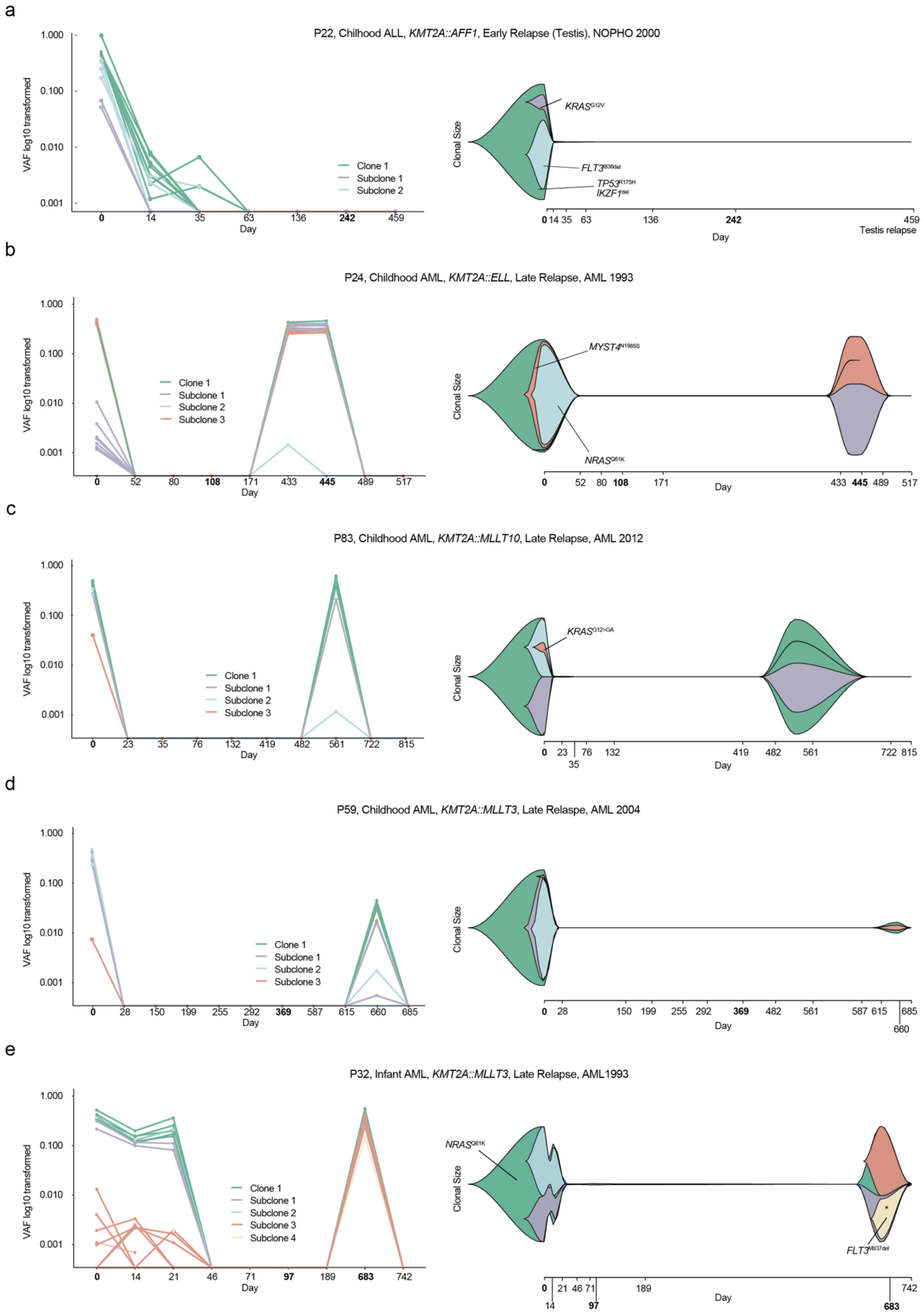

Supplementary Figure 13. Clonal evolution of ALL and AML relapse patients. a-e. Clonal evolution depicted by linear diagrams and fish plots for relapse patients. Longitudinal samples

are on the y-axis, and clonal size on the x-axis. Clones are depicted in different colors. Mutations in the enriched pathways are shown. The VAF is log10 transformed. The * indicates that we cannot determine which clone the subclone belongs to. **a**, Child with a testis relapse at day 459, and the BM did not contain detectable leukemia cells (P22). At least three clones were present at diagnosis, each containing different driver gene mutations. **b,** A child with AML (P24) where the diagnostic *NRAS*$^{\mathrm{Q16K}}$-clone is lost while the *MYST4*^{N1985S}-clone is maintained at relapse. This patient also had a subclonal *MYST4* ^{S1402fs} at diagnosis, a *CCND3*$^{\mathrm{A269fs}}$ at both diagnosis and relapse, and a subclonal *WT1*$^{\mathrm{T360\_L361fs}}$ at relapse (not followed longitudinally). This patient later had another relapse and passed. **c,** A child with AML with a subclonal *KRAS*$^{\mathrm{G12>GA}}$ in 8% of the diagnostic cells, which is lost at relapse (P83). **d,** A child with AML where no driver mutations were detected at diagnosis, and where the relapse was detected and treated early (P59). **e,** An infant with AML where the major diagnostic clone had an *NRAS*$^{\mathrm{Q61K}}$ that was lost at relapse; instead, a *FLT3*^{M837del} was detected in 29% of relapse cells (P32).

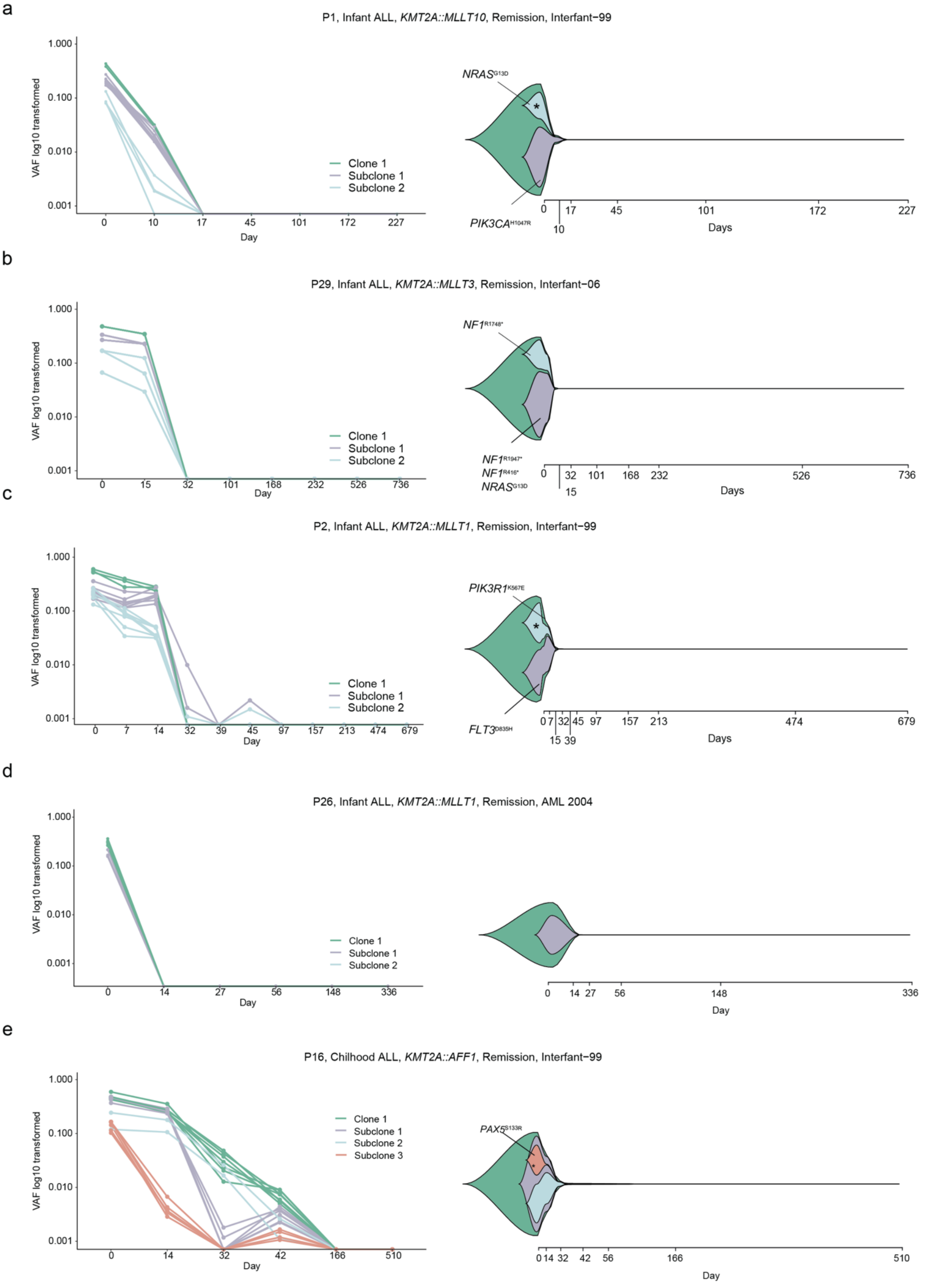

Supplementary Figure 14. Longitudinal evolution of ALL remission cases. a-e. Clonal evolution depicted by linear diagrams and fish plots. Longitudinal samples are on the y-axis

and clonal size on the x-axis. Clones are depicted in different colors. Mutations in the enriched pathways are shown. The * indicates that we cannot say which clone the subclone belongs to. **a,** Infant ALL having both a *PIK3CA* and an *NRAS* mutation, where the *PIK3CA* clone reacted slower to treatment. **b**, Infant ALL with three different *NF1*-mutations, one of them being in a separate clone. **c,** Infant ALL having both a *PIK3R1* and an *FLT3* mutation, where the *FLT3* clone reacted slower to treatment. **d,** Infant with ALL, who was treated according to the NOPHO AML 2004 protocol. **e,** Patient who just turned one, treated on the Interfant-99 protocol instead of NOPHO-2000. The red clone containing a $PAX5^{S133R}$ seems more sensitive to treatment than the blue and lilac clones.

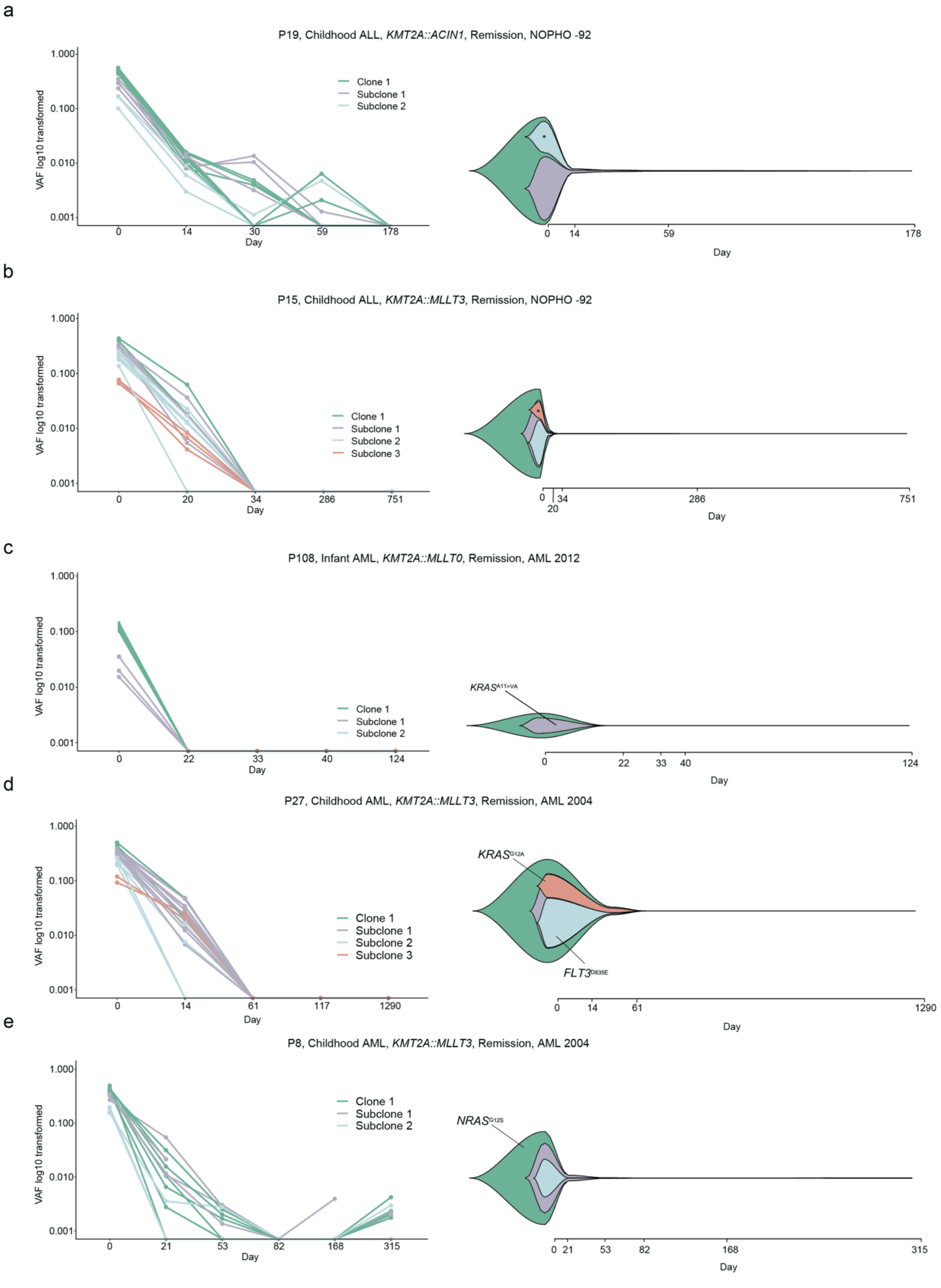

Supplementary Figure 15. Clonal evolution of ALL and AML remission cases. a-c. Clonal evolution depicted by linear diagrams and fish plots. Longitudinal samples are on the y-axis

and clonal size on the x-axis. Clones are depicted in different colors. Mutations in the enriched pathways are shown. The * indicates that we cannot determine which clone the subclone belongs to. **a,** Case with *KMT2A::ACIN1* that lacked additional known driver mutations at diagnosis. **b,** Case treated with NOPHO-1992, which entered remission by day 34. **c,** An AML with a low blast count at diagnosis with a diagnostic *KRAS*$^{\text{A11>VA}}$. **d,** Child with AML with diagnostic *KRAS*$^{\text{G12A}}$ and *FLT3*$^{\text{D835E}}$ subclones. **e,** A child with AML where the major diagnostic clone contained an *NRAS* mutation.

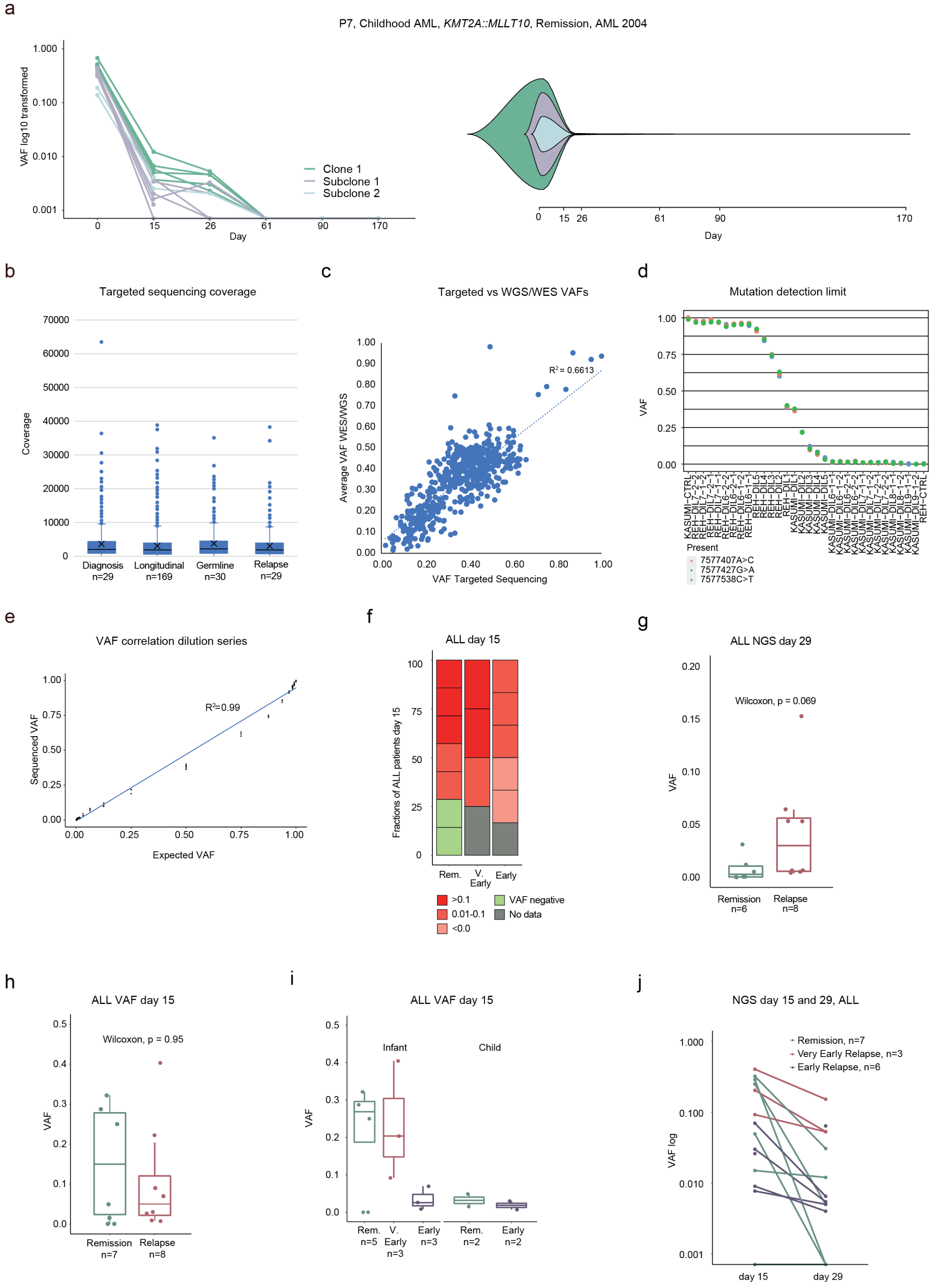

Supplementary Figure 16. Longitudinal targeted deep-sequencing data. **a**, A child with AML and no additional diagnostic driver mutations. **b,** Targeted sequencing coverage for

samples at diagnosis, germline, longitudinal and at relapse. Each point represents one sample. The average is shown by a line and the median by a cross. **c,** Correlation between WGS/WES VAF and next generation/target sequencing (NGS) of SNVs and INDELs detected at diagnosis and relapse. The Pearson correlation coefficient is depicted. **d**, VAF of the three *TP53*-mutations in the investigated samples in dilution order, from highest to lowest VAF. **e,** Correlation between expected VAF (x-axis) and the VAF from our sequencing (y-axis) for the dilution series. For figure **c-e**, source data are provided as a Source Data file. **f,** Stacked plots showing measurable disease in ALL at day 15, grouped into remission and relapse cases. **g,** Box plots showing the VAF at day 29 (end of induction) for ALL remission and relapse cases. **h,** Box plot showing the VAF at the day equivalent to MRD (measurable residual disease) day 15 for the ALL cases divided into remission and relapse cases. **i,** Box plot showing the VAF at the day equivalent to MRD day 15 for the ALL cases divided into infants and children, remission, very early, and early relapse. No significant difference was seen between the groups. The y-axis shows the frequency of the measured VAFs and each line represents a patient. **j,** Graph showing how the maximum VAF per sample changes from day 15 to the end of induction divided into relapse time. For figure **f-j**, source data are provided as a Source Data file.

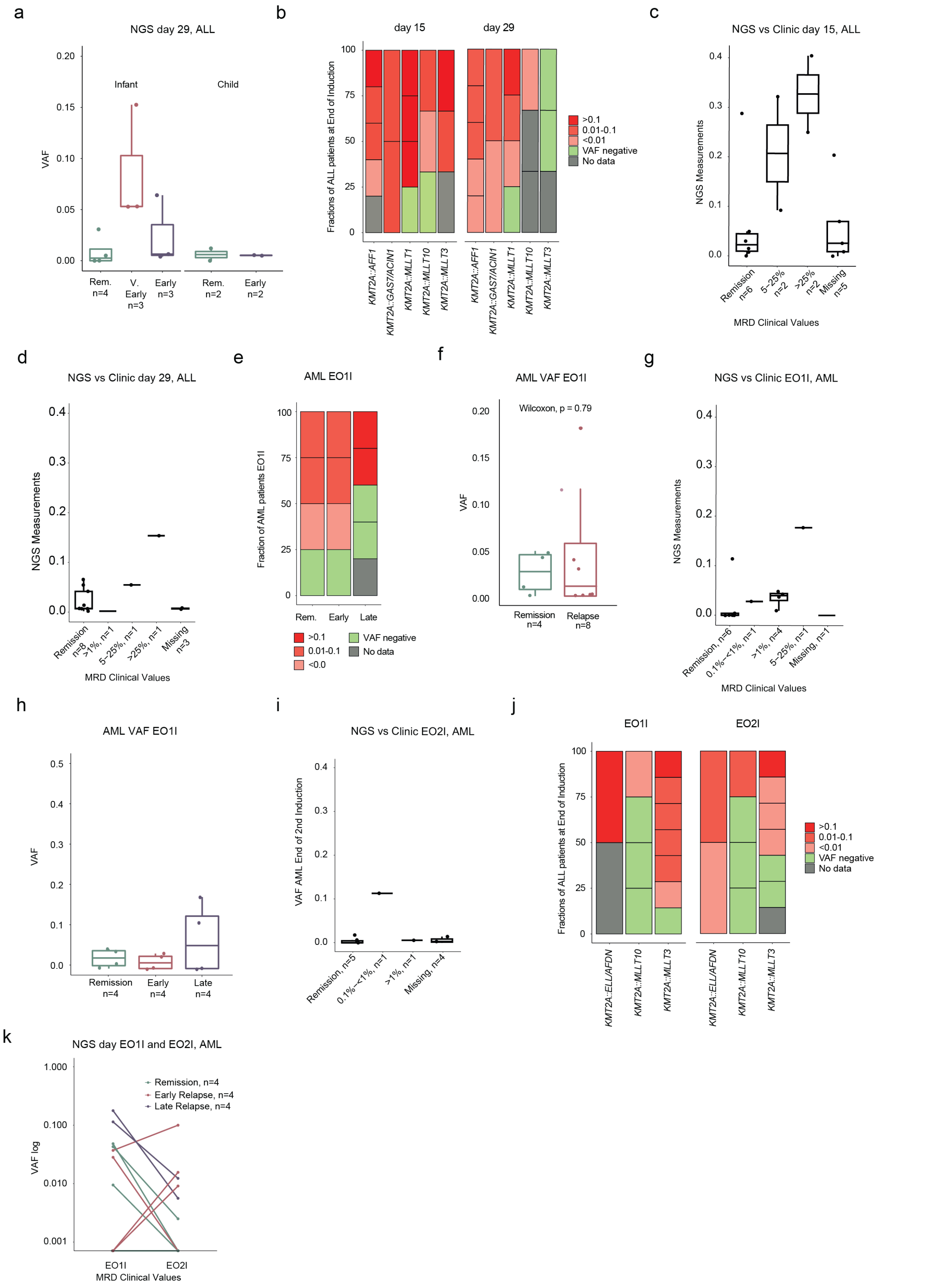

Supplementary Figure 17. Longitudinal targeted deep-sequencing. a, Box plot showing the VAF at the day equivalent to MRD day 29 for the ALL cases divided into infants and children,

remission, very early, and early relapse. No significant difference was seen between the groups. The y-axis shows the frequency of the measured VAFs and each line represents a case. **b,** Stacked plots showing measurable disease in ALL at days 15 and 29 based on the *KMT2A*-rearrangement. The y-axis shows the fraction of cases with a certain VAF as indicated by the color, and each line represents a patient. **c,** Box plots comparing the clinical MRD measures (y-axis) and the measured VAF (x-axis) at day 15 for ALL cases. **d.** Box plots comparing the clinical MRD measures (y-axis) and the measured VAF (x-axis) at day 29 for ALL cases. **e,** Stacked plots showing measurable disease in AML cases at the end of first induction, divided into time to relapse. **f,** Box plots showing the VAF at the end of the first induction for AML remission and relapse patients. **g,** Box plots comparing the clinical MRD measures (y-axis) and the measured VAF (x-axis) after the first induction (EO1I) for AML patients. **h,** Box plot showing the VAF after the first induction (EO1I) for the AML cases divided into remission and relapse patients. **i,** Box plots comparing the clinical MRD measures (y-axis) and the measured VAF (x-axis) after the second induction (EO2I) for AML cases. **j,** Stacked plots showing measurable disease in AML cases at EOI1 and EOI2 based on the *KMT2A*-rearrangement. The y-axis shows the fraction of cases with a certain VAF and each line represents a patient. **k,** Graph showing how the maximum VAF per sample changes from the end of first induction to the end of second induction divided into relapse time. For figure **a-k**, source data are provided as a Source Data file.

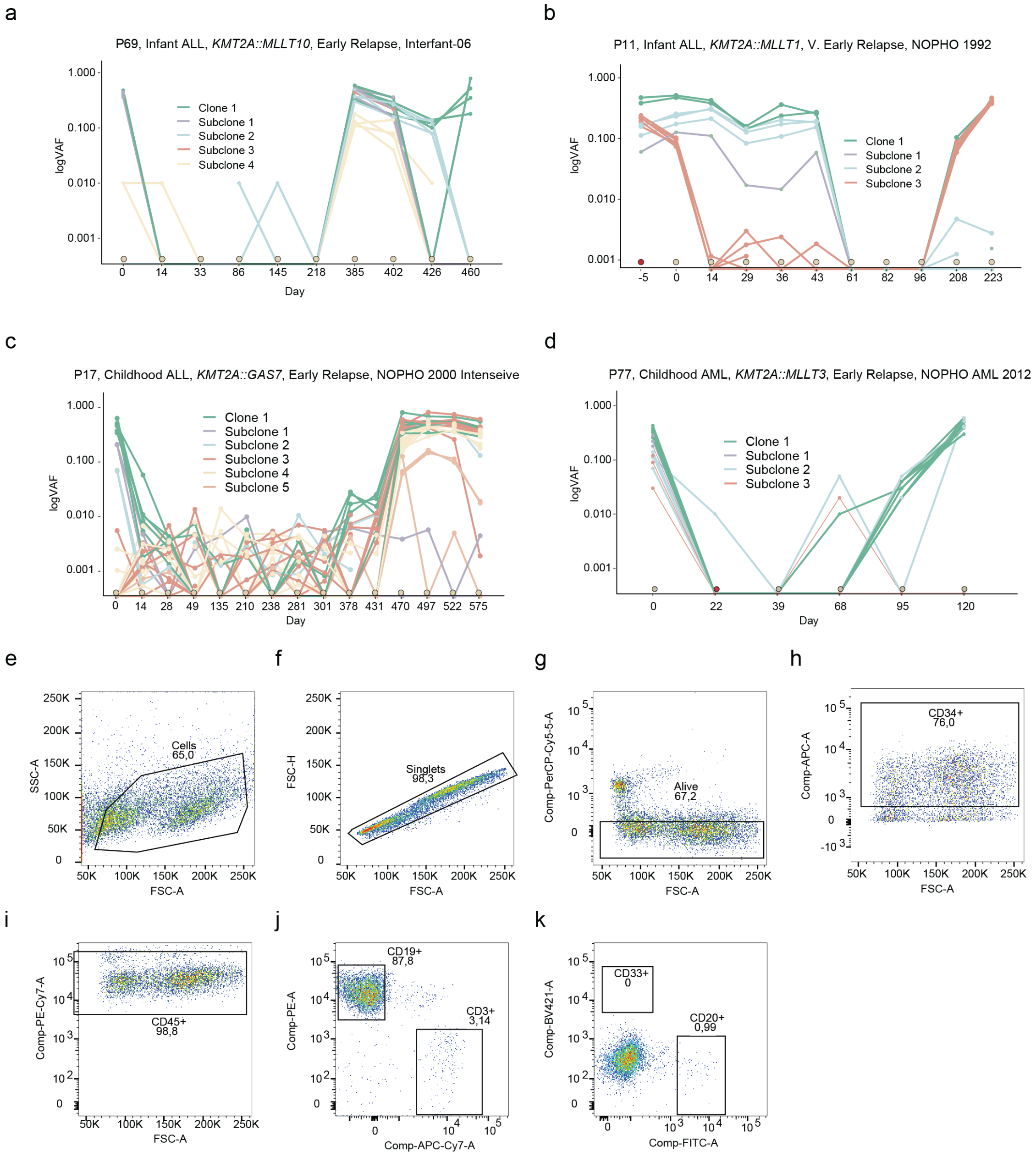

Supplementary Figure 18. Clonal evolution during treatment uncovers unique clonal responses and single-cell sorting. a-d. Clonal evolution depicted by linear diagrams for the cases in main Figure 4. Longitudinal samples are on the y-axis and the clonal size on the x-axis. Clones are depicted in different colors. Mutations in the enriched pathways are illustrated. **e-k,** FACS plots showing the sorting layout for the single cells.
