# Supplementary material for: The genomic landscape of relapsed infant and childhood KMT2A-rearranged acute leukemia
Source: Nat Commun. 2025 Oct 8;16:8964. doi: 10.1038/s41467-025-64190-8 (PMC12508131; doi:10.1038/s41467-025-64190-8)
Supplement: Supplementary file 2 — Description of Additional Supplementary Files [file 41467_2025_64190_MOESM2_ESM.pdf]

Description of Additional Supplementary Files

Supplementary Data 1
Description: Clinical Data.

Supplementary Data 2
Description: WES & WGS Coverage.

Supplementary Data 3
Description: Mutations and Clonal Patterns.

Supplementary Data 4
Description: SNV & INDEL.

Supplementary Data 5
Description: Copy number alterations.

Supplementary Data 6
Description: SV.

Supplementary Data 7
Description: Enriched pathways at relapse.

Supplementary Data 8
Description: Coverage of IKZF1 and TP53 in WGS and WES.

Supplementary Data 9
Description: Detection of Relapse Specific Variants.

Supplementary Data 10
Description: ValidationCohort.

Supplementary Data 11
Description: Mutational Signatures.

Supplementary Data 12

Description: Samples.

Supplementary Data 13

Description: Coverage targeted sequencing.

Supplementary Data 14

Description: MRD.

Supplementary Data 15

Description: Single cell analysis.

Supplementary Data 16

Description: Primers for longitudinal and single cell sequencing.

Supplementary Data 17

Description: Single cell sorting antibodies.

Supplementary Data 18

Description: Functional Assay.
