# Supplementary material for: The genomic landscape of relapsed infant and childhood KMT2A-rearranged acute leukemia
Source: Nat Commun. 2025 Oct 8;16:8964. doi: 10.1038/s41467-025-64190-8 (PMC12508131; doi:10.1038/s41467-025-64190-8)
Supplement: Supplementary file 4 — Reporting Summary [file 41467_2025_64190_MOESM4_ESM.pdf]

nature portfolio

Corresponding author(s): Anna Hagström-Andersson

Last updated by author(s): Apr 22, 2025

Reporting Summary

Nature Portfolio wishes to improve the reproducibility of the work that we publish. This form provides structure for consistency and transparency in reporting. For further information on Nature Portfolio policies, see our Editorial Policies and the Editorial Policy Checklist.

Statistics

For all statistical analyses, confirm that the following items are present in the figure legend, table legend, main text, or Methods section.

n/a	Confirmed	
☐	☒	The exact sample size (n) for each experimental group/condition, given as a discrete number and unit of measurement
☐	☒	A statement on whether measurements were taken from distinct samples or whether the same sample was measured repeatedly
☐	☒	The statistical test(s) used AND whether they are one- or two-sided<br>*Only common tests should be described solely by name; describe more complex techniques in the Methods section.*
☐	☒	A description of all covariates tested
☐	☒	A description of any assumptions or corrections, such as tests of normality and adjustment for multiple comparisons
☐	☒	A full description of the statistical parameters including central tendency (e.g. means) or other basic estimates (e.g. regression coefficient) AND variation (e.g. standard deviation) or associated estimates of uncertainty (e.g. confidence intervals)
☐	☒	For null hypothesis testing, the test statistic (e.g. F, t, r) with confidence intervals, effect sizes, degrees of freedom and P value noted<br>*Give P values as exact values whenever suitable.*
☒	☐	For Bayesian analysis, information on the choice of priors and Markov chain Monte Carlo settings
☒	☐	For hierarchical and complex designs, identification of the appropriate level for tests and full reporting of outcomes
☐	☒	Estimates of effect sizes (e.g. Cohen's d, Pearson's r), indicating how they were calculated

Our web collection on statistics for biologists contains articles on many of the points above.

Software and code

Policy information about availability of computer code

Data collection	not applicable
Data analysis	R version 4.4.0, was used for statistical calculations, using the built-in functions, MutationalPatterns_3.14.0 was used for analysis of mutational signatures. survival_3.5-8 was used for Kaplan-Meier estimations.

For manuscripts utilizing custom algorithms or software that are central to the research but not yet described in published literature, software must be made available to editors and reviewers. We strongly encourage code deposition in a community repository (e.g. GitHub). See the Nature Portfolio guidelines for submitting code & software for further information.

Data

Policy information about availability of data

All manuscripts must include a data availability statement. This statement should provide the following information, where applicable:

- Accession codes, unique identifiers, or web links for publicly available datasets
- A description of any restrictions on data availability
- For clinical datasets or third party data, please ensure that the statement adheres to our policy

The sequencing data generated in this study is deposited in the European Genome-phenome Archive (EGA). The sequencing data are available under restricted access as it is considered personal data and falls under the General Data Protection Regulation (GDPR), and access can be obtained by upon request from the

corresponding author (Anna Hagström-Andersson) through XXXXXXXX. The raw sequencing data are protected and are not available due to data privacy laws. Source data are provided with this paper and the sequencing data generated in this study are provided in the Supplementary Information/Source Data file.

Research involving human participants, their data, or biological material

Policy information about studies with human participants or human data. See also policy information about sex, gender (identity/presentation), and sexual orientation and race, ethnicity and racism.

Reporting on sex and gender	We do not report sex or gender.
Reporting on race, ethnicity, or other socially relevant groupings	We do not report race, ethnicity, or other groupings.
Population characteristics	We have studied infants and children. All patients have been treated according to the specific leukemia protocols, this is stated in the tables and in material and methods.
Recruitment	Patients with KMT2A-rearrangements and relapse were included if a relapse sample was available with enough material for WGS/WES. For the longitudinal patients, patients were included if we had more than 4 longitudinal samples per patient.
Ethics oversight	Informed consent was obtained according to the Declaration of Helsinki and the study was approved by the local Ethics Committee of Lund University, Sweden.

Note that full information on the approval of the study protocol must also be provided in the manuscript.

Field-specific reporting

Please select the one below that is the best fit for your research. If you are not sure, read the appropriate sections before making your selection.

☒ Life sciences ☐ Behavioural & social sciences ☐ Ecological, evolutionary & environmental sciences

For a reference copy of the document with all sections, see nature.com/documents/nr-reporting-summary-flat.pdf

Life sciences study design

All studies must disclose on these points even when the disclosure is negative.

Sample size	KMT2A-r infants and children from the Nordic countries, the interfant group , and USA were studied. The inclusion critera was sample availability.
Data exclusions	Hypermutated patients were excluded from statistical comparisons
Replication	For the dilutions series, up to to four multiplex PCR replicas were studied. For the WGS findings, we analyzed public data to verify our findings.
Randomization	This is not a clinical study and randomization was not required.
Blinding	Blinding was not relevant as we correlated genetic findings with clinical findings.

Reporting for specific materials, systems and methods

We require information from authors about some types of materials, experimental systems and methods used in many studies. Here, indicate whether each material, system or method listed is relevant to your study. If you are not sure if a list item applies to your research, read the appropriate section before selecting a response.

Materials & experimental systems

n/a	Involved in the study	
☐	☒	Antibodies
☐	☒	Eukaryotic cell lines
☒	☐	Palaeontology and archaeology
☒	☐	Animals and other organisms
☐	☒	Clinical data
☒	☐	Dual use research of concern
☒	☐	Plants

Methods

n/a	Involved in the study	
☒	☐	ChIP-seq
☐	☒	Flow cytometry
☒	☐	MRI-based neuroimaging

Antibodies

Antibodies used	Flag-tag antibody, CD3-APC-CY7, CD19-PE, CD20-AF488, CD33-BV421, CD34-APC, CD45-PE-CY7, IgG2a,κ-APC-CY7,IgG1κ -PE ,IgG2b,κ-AF488, IgG1κ -BV421, IgG1κ -APC, IgG1κ -PE-CY7, 7-AAD. See S Table for full information 17
Validation	Negative controls and isotype controls were used when applicable, and ab were validated by the vendors. S Table 17

Eukaryotic cell lines

Policy information about cell lines and Sex and Gender in Research

Cell line source(s)	REH (CRL-8286) ATCC, Female; Kasumi-1 (ACC-220, male DSMZ), REH (ACC-22,Female,DSMZ)
Authentication	REH (ATCC) was authenticated by STR-analysis (used for drug assays). Kasumi and REH was used for mutational analysis to determine sensitivity in a dilution assay thus authentication is not an issue (DSMZ).
Mycoplasma contamination	Cell lines are routinely tested for mycoplasma and were negative
Commonly misidentified lines (See ICLAC register)	No commonly misidentified cell lines were used in the study.

Clinical data

Policy information about clinical studies
All manuscripts should comply with the ICMJE guidelines for publication of clinical research and a completed CONSORT checklist must be included with all submissions.

Clinical trial registration	not applicable
Study protocol	not applicble
Data collection	not applicble
Outcomes	not applicble

Plants

Seed stocks	na
Novel plant genotypes	na
Authentication	na

Flow Cytometry

Plots

Confirm that:

☒ The axis labels state the marker and fluorochrome used (e.g. CD4-FITC).

☒ The axis scales are clearly visible. Include numbers along axes only for bottom left plot of group (a 'group' is an analysis of identical markers).

☒ All plots are contour plots with outliers or pseudocolor plots.

☒ A numerical value for number of cells or percentage (with statistics) is provided.

Methodology

Sample preparation	Single-cell sorting: Sampies were prepared by taking a punch of frozen cells that were thawed in 10% FBS with DNase I (1mg/ml), and RPMI1640, 1% Penicillin-Streptomycin (Thermo Fisher Scientific), centrifuged for 5min, 350xg at 4°C and resuspended in PBS+2% FBS. Cells were blocked with Human TruStain FcX, according to manufacturer's description and stained for 30min at 4°C with antibodies and isotype controls. Cells were washed and resuspended in PBS+2% and incubated

with 7-AAD for 10min at 4°C before flow analysis.

Instrument	FACS Aria Fusion (BD Biosciences)
Software	FACSDiva v8.0.2 (BD)
Cell population abundance	transduction: More than 90% GFP+ cells. For the sc-sorting, we sorted the major leukemia blast cell population; CD 3+ cells were used as neg ctrls (around 3-5% of cells)
Gating strategy	Transduction: Size, live cells, singlets, GFP+: all gates were>69% of cells in prior gates.<br>Singel-cell: Size, live cells, singlets, and then based on Ab-staining.

☒ Tick this box to confirm that a figure exemplifying the gating strategy is provided in the Supplementary Information.
